# A Point in Physiology

**Published:** 1906-12

**Authors:** C. A. F. Lindorme

**Affiliations:** Atlanta, Ga.


### A POINT IN PHYSIOLOGY.

By C. A. F. LINDORME, M.D.,

Atlanta, Ga.

There is a peculiarity in our anatomy which we do not find mentioned in the books.

It is *the arterial function of the portal vein.*

The blood-tract which bears the name of the portal system acts like a true vein until up to its completion at the fissure through which it enters into the liver. The portal vein, about four inches long, is the ultimate trunk into which is collected the blood-return from the viscera of digestion. It is the capillaries of the abdominal aorta and its branches from which the blood-return takes place. The powerful aorta, on entering the abdomen, spreads in ramifying, and widens, until it represents, in its capillaries, two or three hundredfold the vascular surface of the abdominal start, and then, in the mesentery, the portal system takes up the work in reversed style until, in the portal vein proper, the concentration is completed and all the blood of the abdominal aorta, minus the substances which served for the nourishment of the capillary tissue, is collected again in one single trunk, *the vena portæ.*

At this point, however, the scene changes. Inside the liver the service of the portal vein is of a sudden reversed. It does not concentrate any more, but ramifies again. It could not do otherwise; indeed, all the blood to be collected is collected in the portal vein, and its function, if it is to be at all, must be a reversed one; like in an artery, the vascular surface, by ramification, has to be increased. It is widened out until it ends in capillaries again, the blood of which is collected anew by vascular junction, leaving at last only the hepatic veins and the vena cava inferior by which to reach the heart for hydraulic reinforcement.

It is undeniably a very startling feature. The beats of the heart forfeit their power by having to overcome once the slackening of the blood-current caused by the enormous capillary widening of the vascular surface. There is in our anatomy no similar instance of an overcoming twice. If the rhythm of the heart's beats can not be noticed beyond the capillary region, we dare affirm that, in the portal vein, the whole propulsive power of the heart is annihilated by the mentioned exorbitance of nature.

And we have to state another important indication. There is a contrary movement of the contents of the bile-ducts and of the portal vein and the hepatic artery. In the latter two blood-vessels the movement, with reference to the liver, is from outside inside; in the bile-ducts from inside outside; in the hepatic artery and the portal vein the movement is a supply-movement; in the bile-ducts an excretory one.

This is evidently a very noteworthy feature, which, by the way, we have not seen mentioned in either anatomy or physiology. The movement of the blood in the portal vein, inside the liver, is, after all probability, more of a chemical than a physical nature.

The inferences to be drawn from these facts, hygienically and pathologically, are obvious. They warn not to cripple the digestion therapeutics by leaving the difficulty in the liver circulation out of consideration. It is not surprising that liver troubles are so common. The facilities of circulation which are naturally deficient are artifically not supplemented. Not only dietetically, in eating and drinking, the cramming is not avoided, which sets at naught the chemical efforts of the liver as to the requirements of its various metabolism, but medically the attention is too often limited to the alimentary tract, and the liver-predicament left entirely out of play. The liver fights, theoretically and practically, at altogether too great a disadvantage.

